# The Role of HMGB1 in the Pathogenesis of Type 2 Diabetes

**DOI:** 10.1155/2016/2543268

**Published:** 2016-12-22

**Authors:** Yanan Wang, Jixin Zhong, Xiangzhi Zhang, Ziwei Liu, Yuan Yang, Quan Gong, Boxu Ren

**Affiliations:** ^1^Department of Immunology, Medical School, Yangtze University, Jingzhou 434023, China; ^2^Department of Medicine, University of Maryland School of Medicine, Baltimore, MD 21201, USA; ^3^Department of Medicine, Hospital of Yangtze University, Jingzhou 434000, China

## Abstract

*Significance.* With an alarming increase in recent years, diabetes mellitus has become a global challenge. Despite advances in treatment of diabetes mellitus, currently, medications available are unable to control the progression of diabetes and its complications. Growing evidence suggests that inflammation is an important pathogenic mediator in the development of diabetes mellitus. The perspectives including suggestions for new therapies involving the shift from metabolic stress to inflammation should be taken into account.* Critical Issues.* High-mobility group box 1 (HMGB1), a nonhistone nuclear protein regulating gene expression, was rediscovered as an endogenous danger signal molecule to trigger inflammatory responses when released into extracellular milieu in the late 1990s. Given the similarities of inflammatory response in the development of T2D, we will discuss the potential implication of HMGB1 in the pathogenesis of T2D. Importantly, we will summarize and renovate the role of HMGB1 and HMGB1-mediated inflammatory pathways in adipose tissue inflammation, insulin resistance, and islet dysfunction.* Future Directions.* HMGB1 and its downstream receptors RAGE and TLRs may serve as potential antidiabetic targets. Current and forthcoming projects in this territory will pave the way for prospective approaches targeting the center of HMGB1-mediated inflammation to improve T2D and its complications.

## 1. Introduction

It is reported that there are approximately 10 percent of the adult population suffering from diabetes in the world. More importantly, the incidence of diabetes mellitus is increasing at an alarming rate [1]. T2D, a metabolic disorder formed after a long and complicate pathological process, is characterized by decreased insulin sensitivity and following pancreatic *β*-cell dysfunction [2, 3]. After long-term overnutrition, metabolic balance of body is broken and becomes an origination of insulin resistance. Insulin resistance leads to compensatory increase of insulin secretion and *β*-cell hypertrophy. Long-term overload of work may result in islet *β*-cell dysfunction even death. Several metabolic processes, such as endoplasmic reticulum stress, hypoxia, and lipotoxicity, are all involved in overnutrient-induced metabolic inflammation. Importantly, obesity and obesity-associated inflammation have long been proposed to be responsible for insulin resistance and T2D [4].

T2D has been associated with chronic low-grade inflammation for decades. Given the importance of innate immunity in inflammation, innate immune mediators may play a vital role in the development of this disease. Emerging evidence has indicated that HMGB1, a highly conserved nonhistone nuclear protein that serves as a damage associated molecule pattern molecule, is associated with the pathogenesis of T2D. HMGB1 can signal through receptor for advanced glycation end products (RAGE) and Toll-like receptors (TLRs) to activate nuclear factor-*κ*B (NF-*κ*B) signaling pathway [5, 6] thus contributing to the inflammatory responses in T2D.

In this review we will focus on the pathophysiological connections between HMGB1 and obesity, insulin resistance, and islet dysfunction. The understanding of the role of HMGB1 may provide a new insight into anti-inflammatory therapeutic strategies for T2D.

## 2. Type 2 Diabetes

T2D, also called non-insulin-dependent diabetes mellitus, is characterized by insulin resistance and pancreatic *β*-cell dysfunction, resulting from an unsettled hyperglycemia condition [2, 3]. Insulin resistance runs through the whole process of diabetes. The liver, muscle, and adipose tissue can act as sites of insulin resistance. In order to compensate for insulin resistance, islet *β* cells produce more insulin which may exceed the maximum capability and results in *β* cells failure [7].

Chronic, low-grade adipose tissue inflammation links obesity and insulin resistance, thereby playing a key role in the early phase of T2D. In recent years, the role of inflammation in the pathogenesis of T2D has been extensively studied. It has been shown that the peroxisome proliferator activated receptor (PPAR) *α*/*γ* agonists attenuated insulin resistance in human adipocytes via reducing proinflammatory mediators including interleukin- (IL-) 6, CXC-L10, and monocyte chemoattractant protein (MCP)-1 [8]. Another research reported that insulin significantly reduced several key mediators of inflammatory stress in humans [9]. These studies indicated that anti-inflammatory mechanism might play a role in antidiabetic action. In other words, T2D is an inflammatory disease.

As the two major features of T2D, both insulin resistance and *β*-cell dysfunction are closely related to inflammation. Proinflammatory macrophages in obese adipose tissue and insulin resistance state are main immune cells, and there is a detailed description of this process in an excellent recent review [4]. Proinflammatory factor, tumor necrosis factor- (TNF-) *α*, was the first inflammatory maker that was been suggested to play a role in the development of obesity-induced insulin resistance in the 1990s [10, 11]. In 1993, Hotamisligil et al. [12] demonstrated that adipose tissue of obese rodents secreted TNF-*α*, a potent negative regulator of insulin signaling. TNF-*α* stimulates adipocyte lipolysis contributing to elevated serum free fatty acids (FFAs) concentrations, which can lead to decreased insulin sensitivity. Now, there are many lines of researches illustrating that obesity and obesity-induced insulin resistance are closely linked to inflammation. Recent studies have also identified a number of cellular and molecular players participating in the development of T2D.

## 3. HMGB1

HMGB1, a nuclear protein, was first known for its role in the regulation of gene expression. Recent advances implicated that HMGB1 had alarming activities via activating proinflammatory responses after being passively released by necrotic cells or actively secreted by activated immune cells into the extracellular milieu [13, 14]. HMGB1, as an endogenous danger signal triggering inflammatory responses, appears to play an important role in the pathogenesis of several inflammatory conditions, including sepsis, arthritis, cancer, and autoimmunity diseases.

### 3.1. HMGB1 Biochemistry, Tissue Distribution, and Structure

HMGB1 was extracted and identified in bovine thymus for the first time in 1973 by Goodwin and Johns [15] and was named because of its high migration ability in polyacrylamide gel electrophoresis. According to the molecular weight, sequence similarity, and DNA structure, HMG can be further divided into three families: HMGA, HMGB, and HMGN. HMGB1 is the most abundant HMG protein. HMGB1 is a nonhistone chromosomal binding protein mainly located in the nucleus of most tissues, wherein it binds to DNA and regulates chromatin remodeling, DNA damage repair, and gene transcription [16, 17]. It is highly evolutionarily conserved in vertebrate animals and is widely distributed in lymphoid tissue, brain, liver, lung, heart, spleen, kidney, and other tissues.

HMGB1 molecule is expressed as a single polypeptide chain of 215 amino acids (AA) and is composed of three distinct structural domains: A-box (AA 1–79), B-box (AA 89–162), and the acidic C tail (AA 186–215) [18]. Both A-box and B-box are able to bind to DNA and participate in the folding and twisting of the DNA. The B-box is the functional region of inflammation. It consists of two crucial binding sites for TLR4 and RAGE and thus plays an important role in promoting inflammation. In comparison, the A-box competes with full-length HMGB1 for binding sites and thus induces anti-inflammatory effects [19–21]. One of our studies using recombinant A-box confirmed that the HMGB1 A-box was able to alleviate LPS-induced inflammation in the lung and modulate acute lung injury [22]. The acidic C terminus is enriched with negatively charged aspartic acid and glutamic acid for transcription stimulation.

HMGB1 also undergoes posttranslational modification which determines its bioactivity. For example, there are 3 conserved redox-sensitive cysteines (C23, C45, and C106). The disulfide linkage of C23 and C45 is required for the cytokine-stimulating activity of HMGB1 and C106 must remain in its reduced form as a thiol at the same time [23]. In addition, both the acetylation of HMGB1 within its nuclear localization sequences [24] and the methylation at lysine 42 [25] promote HMGB1 release by mobilizing HMGB1 from the nucleus to the cytoplasm.

### 3.2. HMGB1 Physiologic Function

HMGB1 is widely expressed in mammalian tissues and present in all vertebrate nuclei [26]. It was first discovered as a nuclear protein. Nuclear HMGB1 binds to DNA and regulates a number of key DNA events including V(D)J recombination, gene transcription, DNA replication, and DNA repair [27–31]. It was considered exclusively as a nuclear protein until 1999 that Wang et al. [32] first reported HMGB1 acted as a late inflammatory mediator and was involved in the pathogenesis of sepsis. Afterwards, people began to recognize that HMGB1 played a significant role in the process of inflammation.

HMGB1 can be either actively secreted by stimulated immune cells including activated monocytes, macrophages, mature dendritic cells, natural killer cells, and endothelial cells, or passively released by necrotic and damaged cells [33–36]. When secreted or released into extracellular environment, HMGB1 participates in several processes such as immune response, cell migration, cell differentiation, proliferation, and tissue regeneration. HMGB1 is found to be associated with many inflammatory diseases such as cancer, trauma, arthritis, ischemia reperfusion injury, sepsis, cardiovascular shock, diabetes, and autoimmune diseases [37–41]. In addition, HMGB1 has been shown to be a crucial coordinator of acute inflammation in various stress models [42].

## 4. HMGB1 Mediated Signaling Pathways

The proinflammatory signaling of HMGB1 is mainly transduced by RAGE and TLRs. RAGE was the first receptor identified to be able to bind HMGB1. Nevertheless, the interaction with RAGE alone could not fully explain the complicated effects of HMGB1. Subsequent studies demonstrated that HMGB1 might promote inflammation via interacting with TLRs (TLR2/4/9) and activating their downstream signaling pathways. HMGB1, via interacting with its receptors, ultimately results in the activation of NF-*κ*B and production of proinflammatory cytokines including IL-6, IL-1*β*, and TNF-*α*. Interestingly, the activation of NF-*κ*B pathway could in turn induce the expression of HMGB1 and its receptors, forming a positive feedback loop to sustain inflammatory conditions [43].

In recent studies, HMGB1 has also been suggested to signal through integrin [44], CXCR4 [45, 46], and triggering receptor expressed on myeloid cell 1 (TREM1) [47]. However, the effects of these receptors and their downstream signaling pathways are not completely understood. In this section, we will briefly describe RAGE and Toll2/4/9-mediated signaling.

### 4.1. RAGE

RAGE was first discovered as a cell surface receptor for advanced glycation end products (AGEs) and is now recognized as a receptor for diverse ligands, including HMGB1, *β*-amyloids [48], and S100 proteins [49, 50]. It is a primary receptor for HMGB1 [51].

RAGE is expressed on a various types of cells including monocytes, macrophages, neurons, endothelial cells, and many tumor cells [52, 53]. Knockout of RAGE reduced tumor growth and metastasis [54, 55] and augmented chemotherapy resistance [56]. HMGB1 and RAGE interaction mainly induces CDC42/Rac and mitogen-activated protein kinase (MAPKs) activation, which finally result in NF-*κ*B activation [49, 52, 57]. The activation of CDC42/Rac and MAPKs pathways also promotes chemotaxis, cytokines production [58], endothelial cells activation [59], immune cells maturation, and migration [60, 61].

HMGB1/RAGE binding activates the two canonical MAPKs, extracellular regulated protein kinases (ERK1/2) and p38 MAPK, resulting in the nuclear translocation of NF-*κ*B and transcription of proinflammation cytokine [52, 62, 63]. The HMGB1/RAGE axis has been suggested to contribute to the pathogenesis of many diseases such as acute lung injury [64], preeclampsia [65], diabetes [66], cancer [67, 68], and autoimmune diseases [69].

### 4.2. TLRs

TLRs are members of the type-1 transmembrane glycoprotein family which can bind extracellular ligands and act as a transducer to sponsor proinflammatory signaling through ectodomain leucine-rich repeated (LRR) sequences and cytoplasmic Toll/interleukin-1 receptor (TIR) domain [70]. TLRs are characterized as one species of pattern recognition receptors to recognize several danger signals, including pathogen associated molecular patterns (PAMPs) and damage associated molecular patterns (DAMPs), and thus are involved in innate immune responses against infection and injury [71]. So far, 13 members of TLRs have been discovered. Among those, HMGB1 can interact with TLR2, TLR4, and TLR9 [72–74]. HMGB1 signals through these three TLRs and activates myeloid differentiation factor 88- (MyD88-)-dependent pathway to activate the NF-*κ*B and interferon regulatory factor (IRF) pathways. Studies suggested an intramolecular Cys23–Cys45 fragment and Cys106 residue within the HMGB1 were required for TLR4 binding and activation [75, 76], while HMGB1-TLR2 structural basis needs to be further investigated. It was well elucidated that HMGB1 binding to lipopolysaccharide contributed to the transfer of the complex to CD14 and, subsequently, to TLR4/MD2 complex [77–79]. Furthermore, it was researched that the TLR4/MD2 receptor system specially recognized the disulfide HMGB1, and this interaction was of great significance for HMGB1-mediated inflammation [80]. TLR9 is a member of the TLR family located in the endoplasmic reticulum-Golgi intermediate compartment [81]. It was reported that TLR9 was a receptor for unmethylated CpG-DNA [82]. HMGB1 acted as a CpG-ODN-binding protein that promoted cytokine release through interacting with TLR9 and signaling through downstream pathways involving MyD88 and NF-*κ*B molecules [83]. Moreover, HMGB1-CpG-DNA complex was responsible for the redistribution of TLR9 to early endosomes and TLR9-mediated cytokine production [73, 83].

## 5. Role of HMGB1 in Type 2 Diabetes

**Figure 1:**
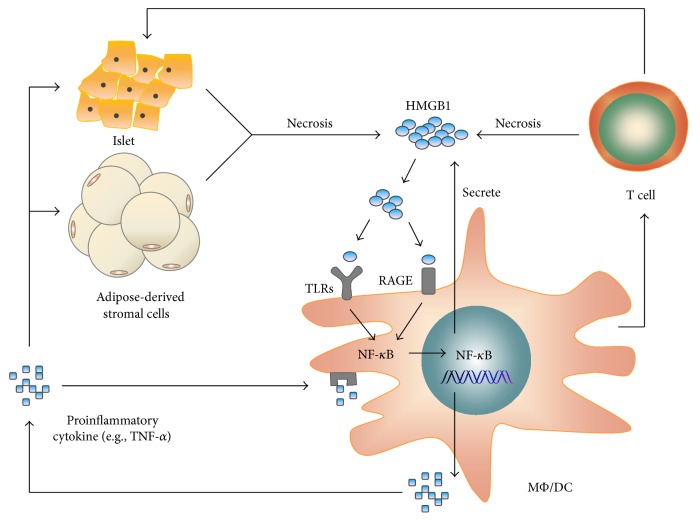
Potential involvement of HMGB1 in type 2 diabetes. Early inflammation in adipose tissue and pancreatic islets leads to the necrosis of adipose-derived stromal cells and islet cells. Necrotic cells release HMGB1, activating TLRs and RAGE on macrophages and dendritic cells. Activation of TLRs and RAGE leads to the translocation of NF-*κ*B into nucleus to promote the expression of inflammatory gene, which contributes to the secretion of proinflammatory cytokine, including HMGB1. In addition, activated macrophages and dendritic cells actively secrete HMGB1, which, in turn, exacerbate the necrosis of adipose tissue and pancreatic islets.

HMGB1, as a late mediator of inflammation, has been proposed to be a significant mediator in the pathogenesis of a variety of diseases including T2D (Figure 1). The correlation between level of HMGB1 and diabetic complications has been reported. Increased levels of HMGB1 have been reported in both diabetic patients and animal models. Pachydaki et al. [84] and Yu et al. [85] observed an elevated expression of this protein in the retinas of diabetic patients and rat models with retinopathy. Moreover, Dasu et al. [86] showed that circulating levels of HMGB1 were higher in type 2 diabetic patients than control, and the same phenomenon was observed by Škrha et al. [87] Hagiwara et al. [88] demonstrated that hyperglycemia, induced by infusion of glucose in a rat model, was associated with elevated serum HMGB1 levels. In addition, metformin, a first-line antidiabetic drug, was also known to have anti-inflammatory effects. Tsoyi et al. [89] indicated that metformin significantly decreased HMGB1 expression in LPS-treated RAW264.7 cells. Another report by Zhang et al. [90] demonstrated that metformin protected against hyperglycemia-induced cardiomyocyte injury by inhibiting the expression of RAGE and HMGB1.

### 5.1. HMGB1 and Adipose Tissue Inflammation

Clinical and experimental researches showed that inflammation with the infiltration of macrophages occurred in the adipose tissue [91]. Although each cell type within the adipose tissue (such as preadipocyte, adipocyte, T cell, dendritic cell, and macrophage) has a contribution to obesity-induced inflammation and insulin resistance [92], macrophage is the primary source of inflammatory effectors [93, 94]. Macrophages in the adipose tissue can switch from an anti-inflammatory “M2” (alternatively activated) state to a proinflammatory “M1” (classically activated) state [95, 96]. M1 macrophages exert proinflammatory effects through the secretion of cytokines TNF-*α*, IL-1*β*, and IL-6 [97], whereas M2 macrophages produce anti-inflammation cytokines such as IL-10 in contrast [98]. The imbalance of M1/M2 leads to an increased secretion of proinflammatory factors such as TNF-*α* [99]. Moreover, Vandanmagsar et al. [100] showed that macrophage inflammation was also mediated by an excess of fatty acids (FAs) via TLR or NOD-like receptor family, one of which is the NOD-like receptor family, the pyrin domain containing 3 (NLRP3) dependent pathway. However, there was one study showing that endotoxin-free, free fatty acids did not have the ability to activate TLR4 and thereby cause inflammation [101]. More importantly, they showed that some polyunsaturated fatty acids exerted an anti-inflammatory action in human adipose tissue and adipocytes. That might mean that HMGB1 was an even more important TLR4 ligand if FFAs indeed were unable to signal via TLR4.

A growing number of studies elucidated a role of HMGB1 in adipose tissue, such as activating proinflammatory macrophages. Recently, a study among obese children showed that obesity was positively correlated with increased HMGB1 serum levels and was related to a number of proinflammatory cytokines, such as IL-6 and TNF-*α* [102]. Another study indicated that mice receiving the anti-HMGB1 antibody gained less weight than the control animals [103]. However, anti-HMGB1 treatment only reduced the expression of TNF-*α* and MCP-1 in the liver, but not in the adipose tissue [103]. They were unable to detect an improvement of inflammation in high fat-induced adipose tissue after anti-HMGB1 antibody treatment. Kanellakis and colleagues demonstrated that administration of anti-HMGB1 antibody led to a reduction in macrophage content of atherosclerotic lesions in Apo-E deficient mice [104]. Indeed, in a recent study, Song [105] and colleagues found that high fat feeding induced expression of HMGB1 in the liver and adipose tissue, while genetic deficiency of HMGB1 receptor RAGE prevented the effects of high fat diet on energy expenditure, weight gain, adipose tissue inflammation, and insulin resistance.

The role of HMGB1 on macrophages has been clearly described and stimulation of macrophages with HMGB1 induced production of proinflammatory cytokines [53] which may lead to an increase of adipose tissue inflammation and insulin resistance. Yu et al. [72] showed that anti-TLR2 antibodies could reduce the binding of HMGB1 on the cell surface of murine macrophage-like RAW 264.7 cells, leading to a reduction in inflammation. Moreover, Yang et al. [75] reported that HMGB1 bound specifically to TLR4, leading to TNF release from macrophages. Inhibition of HMGB1/TLR4 binding with neutralizing anti-HMGB1 mAb prevented HMGB1-induced cytokine release. These findings suggested that HMGB1 might have an important role in the initiation and development of adipose tissue inflammation.

### 5.2. HMGB1 and Insulin Resistance

Insulin resistance refers to decreased capability of insulin to promote glucose uptake. In simple terms, insulin resistance is a condition in which cells fail to appropriately respond to circulating insulin [106, 107]. All of the peripheral tissues such as liver, muscle, and adipose tissue can act as a site of insulin resistance. It has been shown that adipose tissue macrophage infiltration was associated with an elevated level of serum insulin [108]. Therefore, macrophage-mediated inflammatory response might be a critical contributor of insulin resistance.

The occurrence and development of insulin resistance are closely related to the two signaling pathways: Jun N-terminal kinase (JNK) and I*κ*B kinase complex (IKK)*β*/NF-*κ*B [109]. The JNK is known to play a role in regulating the development of obesity-induced insulin resistance. The activation of the JNK decreased insulin sensitivity and this related in part to the phosphorylation of insulin receptor substrate- (IRS-) 1 on the serine 307 and 310 residues [110, 111]. Proinflammation molecules activate JNK to promote serine phosphorylation of IRS-1 and IRS-2, a process closely related to insulin resistance. Furthermore, knockout of JNKs ameliorated obesity-induced insulin resistance [112, 113]. However, there was no clear evidence showing that HMGB1 was involved in JNK activation. On the contrary, the NF-*κ*B signaling pathway is closely related to HMGB1.

Indeed, NF-*κ*B is a major factor in the regulation of inflammation [114, 115]. In resting cells, NF-*κ*B and I*κ*B complex locates in the cytoplasm and remains inactive. As the inhibitor of the NF-*κ*B, I*κ*B binds to NF-*κ*B through its specific ankyrin repeat motif in C-terminal. This binding covers the nuclear localization sequence (NLS) of NF-*κ*B and thus prevents its transfer to the nucleus. When the cells are stimulated by extracellular signals, IKK is activated. IKK*β*, the catalytic subunit of IKK, phosphorylates I*κ*B. Following the degradation of I*κ*B, the NLS of NF-*κ*B is exposed and the free NF-*κ*B translocates to the nucleus, where it regulates the expression of multiple inflammatory genes including TNF-*α*, IL-1*β*, IL-2, and IL-6.

Increased NF-*κ*B activity has been observed in an obese animal model [116]. Blocking NF-*κ*B protected high fat diet mice from insulin resistance [117]. NF-*κ*B signaling pathway could be activated by pattern recognition receptors like TLRs and RAGE, both of which have been shown to interact with HMGB1. This suggested that HMGB1 might play a critical role in insulin resistance through NF-*κ*B signaling. In a number of human studies with obese state or T2D, circulating level of HMGB1 was higher and positively correlated with homeostasis model assessment-insulin resistance (HOMA-IR) [102, 118–120]. In a further research of Dasu and colleagues, the amount of HMGB1 was elevated in human type 2 diabetic subjects and positively correlated with TLR2 and TLR4, BMI, and HOMA-IR. In addition, the expression of MyD88 and NF-*κ*B p65 was increased [86]. The activation of TLR-MyD88-NF-*κ*B signaling resulted in elevated levels of cytokines [86]. Soon afterwards, Chen et al. [121] found that the expression of HMGB1 and NF-*κ*B and TNF-*α*/vascular endothelial growth factor (VEGF) were significantly upregulated in type 2 diabetic retinas and in high glucose treated ARPE-19 cells, compared to their respective counterparts. HMGB1 blockade significantly alleviated NF-*κ*B activity and VEGF secretion in ARPE-19 cells stimulated with high glucose. Chen et al. [122] indicated that HMGB1 was significantly upregulated by high glucose via NF-*κ*B signaling in vivo and in vitro, in an association with an elevation of proinflammatory cytokines. HMGB1 inhibition reduced the upregulation of proinflammatory cytokines in response to high glucose. Hence, HMGB1 might be involved in the development of insulin resistance via activating NF-*κ*B signaling and associated with a raised expression of proinflammation mediators.

### 5.3. HMGB1 and Pancreatic Islet Inflammation

Recently, a number of studies suggested that there was a sustained inflammatory milieu in pancreatic islets in the process of T2D, owing to increased cytokine or chemokine production and immune cell infiltration. Recent studies on human islets with T2D demonstrated that the combination of hyperglycemia and elevated FFAs induced a more efficient proinflammatory phenotype, mainly via TLR, MyD88, and interleukin-1 receptor (IL-1R) I signals [123]. Studies in mouse showed that elevated levels of circulating saturated FAs activated inflammatory processes within islets via TLR4/MyD88 pathway. *β* cells produced chemokines and recruited CD11b(+)Ly-6C(+) M1-type proinflammatory monocytes/macrophages to the islets [124]. IL-1 plays a critical role in the islet inflammation. Type 2 diabetic GK rat displayed an increased expression of IL-1*β*, and specific blockade of IL-1 activity by the IL-1 receptor antagonist (IL-1Ra) was associated with reduced inflammatory cytokines/chemokines in GK islets in vitro and in vivo when exposed to metabolic stress [125]. Meanwhile, TLR2 and TLR4 and NLRP3 inflammasome also play a role in islet inflammation and islet dysfunction [126]. About ten years ago, Steer and colleagues elucidated that IL-1 combined with HMGB1 promoted islet inflammation and *β*-cell death, which provided a proof for HMGB1 in the inflammation of pancreatic islet cells [127]. However, there was no further study of HMGB1 involvement in islet inflammation later. Instead, there were studies focusing on the role of HMGB1 in islet transplantation [128–133]. HMGB1 inhibition by anti-IL-6R antibody, HMGB1 A-box, NF-*κ*B inhibitor, or the Na(+)/Ca(2+) exchanger inhibitor was able to ameliorate inflammatory responses and islet survival after islet transplantation by reducing the amount of innate immune cells and cytokines like TNF-*α* and interferon- (IFN-) *γ* [129–132]. And blockade of HMGB1 with anti-HMGB1 monoclonal antibody (mAb, 2g7) inhibited inflammatory response by reducing TNF-*α* and IL-1*β* production and thereby improved islet viability after islet cells transplantation [134].

### 5.4. HMGB1 and *β*-Cell Death

Pancreatic *β*-cell death is a major feature of the late stage of T2D. It is a consequence of insulin resistance and *β*-cell overcompensation. In a clinical study of T2D, the reduction of *β*-cell mass was observed in the pancreatic tissue sections [2]. It was reported that decreased *β*-cell mass in T2D was attributed to pancreatic *β*-cell apoptosis and to *β*-cell dedifferentiation [135]. Furthermore, *β*-cell dysfunction and apoptosis were associated with islet inflammation. Richardson et al. [136] suggested that a persistent, low-grade enteroviral infection of islet endocrine cells might induce functional changes that contribute to the recruitment of macrophages in some patients with T2D. This article gave evidence that macrophage infiltration was involved in *β*-cell dysfunction and loss in T2D. In addition, saturated FAs activated inflammatory processes within islets via activating TLR4/MyD88 pathway and engendering chemokines [124]. These results demonstrated that islet inflammation resulted in *β*-cell dysfunction and death. Alternately, HMGB1 played an important role in the process of *β*-cell apoptosis according to reports. Li et al. [13] demonstrated that TLR4 was the main receptor of HMGB1 on *β* cells and HMGB1 might signal through TLR4 to selectively damage *β* cells rather than *α* cells in type 1 diabetes mellitus. Another study in NOD mice indicated that HMGB1 could be passively released from damaged pancreatic *β* cells and actively secreted by islet infiltrated immune cells. Blockade of HMGB1 inhibited diabetes development in younger NOD mice [137]. These two studies together suggested that extracellular HMGB1 led to islet *β* cells damage via binding to TLR4 on *β* cells. Necrotic islet *β* cells, in turn, released HMGB1 to accelerate cell damage, creating a vicious loop. In addition, HMGB1 and RAGE interaction induced islet cells apoptosis and progressive *β*-cell loss by inducing oxidative stress in type 2 diabetic rats [138]. One study showed that high concentration of insulin resulted in increased extracelluar HMGB1 and induced the apoptosis of rat ovarian granulosa cells in vitro [139]. It has also been reported that HMGB1 could directly stimulate pancreatic *β* cells to secrete insulin [120].

## 6. Conclusion Remarks

HMGB1, an ubiquitously expressed and evolutionarily conserved chromosomal protein, has diverse roles in mediating inflammation. Increasing evidence indicates HMGB1 is a vital mediator in the onset and progression of diabetes. Elevated level of HMGB1 has been found in serum and islets as well as other tissues like adipose, liver, and muscle from diabetic patients and animal models. Additionally, increased expression levels of RAGE and TLRs have also been reported in diabetes, and these receptors are critically involved in the induction of proinflammatory cytokines. The functional association among HMGB1, RAGE, and TLRs augments the inflammation in the course of T2D including obesity-induced inflammation, insulin resistance, and islet inflammation. Therefore, blockade of HMGB1 and its receptors represents a promising therapeutic approach for controlling inflammation in T2D.
